# Utility of diffusion-weighted and contrast-enhanced magnetic resonance imaging in diagnosing and differentiating between high- and low-grade uterine endometrial stromal sarcoma

**DOI:** 10.1186/s40644-019-0247-z

**Published:** 2019-09-12

**Authors:** Yen-Ling Huang, Shir-Hwa Ueng, Kueian Chen, Yu-Ting Huang, Hsin-Ying Lu, Koon-Kwan Ng, Ting-Chang Chang, Chyong-Huey Lai, Gigin Lin

**Affiliations:** 1 Department of Medical Imaging and Intervention, Chang Gung Memorial Hospital at Linkou, 5 Fuhsing St., Guishan, Taoyuan, Taiwan 33382; 2 Imaging Core Laboratory, Institute for Radiological Research, Chang Gung Memorial Hospital at Linkou and Chang Gung University, 5 Fuhsing St., Guishan, Taoyuan, Taiwan 33382; 3 Department of Pathology, Chang Gung Memorial Hospital at Linkou and Chang Gung University, 5 Fuhsing St., Guishan, Taoyuan, Taiwan 33382; 4 Department of Obstetrics and Gynecology and Gynecologic Cancer Research Center, Chang Gung Memorial Hospital at Linkou and Chang Gung University, 5 Fuhsing St., Guishan, Taoyuan, Taiwan 33382; 5 Department of Diagnostic Radiology, Chang Gung Memorial Hospital at Keelung, 222, Maijin Rd, Keelung, Taiwan 20401; 6 Clinical Metabolomics Core Laboratory, Chang Gung Memorial Hospital at Linkou, 5 Fuhsing St., Guishan, Taoyuan, Taiwan 33382; 7 Clinical Trial Center, Chang Gung Memorial Hospital at Linkou and Chang Gung University, 5 Fuhsing St., Guishan, Taoyuan, Taiwan 33382

**Keywords:** Endometrial stromal sarcoma, Uterine leiomyoma, Magnetic resonance imaging, Diffusion-weighted imaging, Accuracy, Grading

## Abstract

**Background:**

Endometrial stromal sarcoma (ESS) is a rare uterine malignancy that features different prognoses for its high- and low-grade subtypes. We investigated the diagnostic accuracy of magnetic resonance (MR) imaging in diagnosing and differentiating between high- and low-grade ESS.

**Methods:**

We retrospectively reviewed the preoperative pelvic MR images of consecutive patients who received histologically confirmed diagnoses of high-grade ESS (*n* = 11) and low-grade ESS (*n* = 9) and T2-hyperintense leiomyoma (*n* = 16). Two radiologists independently evaluated imaging features in T1-, T2-, and diffusion-weighted and contrast-enhanced MR images. Statistical analysis included Mann-Whitney tests and Fisher’s exact test, with sensitivity, specificity and diagnostic accuracy of imaging features.

**Results:**

High-grade ESS was associated with significantly more extensive necrosis and hemorrhage and distinct feather-like enhancement compared with low-grade ESS (*P* < .05 for all). The feather-like enhancement pattern yielded a diagnostic accuracy of 95%, sensitivity of 91%, and specificity of 100% in differentiating high-grade from low-grade ESS. This imaging characteristic was significantly superior to the necrosis (80%, *P* = .033) or hemorrhage (75%, *P* = .007). Both high- and low-grade ESS demonstrated T2 hypointense bands, marginal nodules, intratumoral nodules, and worm-like intra-myometrial nodules, and their tumor apparent diffusion coefficient (ADC) values were significantly lower than those of T2-hyperintense leiomyomas (*P* < .001).

**Conclusions:**

Diffusion-weighted MR imaging is useful in diagnosing ESS against T2-hyperintense leiomyomas, and contrast enhancement aids in further differentiating between high- and low-grade ESS.

**Electronic supplementary material:**

The online version of this article (10.1186/s40644-019-0247-z) contains supplementary material, which is available to authorized users.

## Background

Uterine sarcomas are rare but aggressive, and they contribute to 3–7% of all uterine malignancy cases and to 26% of total cancer related mortality [1–3]. Endometrial stromal sarcoma (ESS) accounts for 10–21% of all uterine sarcoma cases, and the clinical presentation of ESS mimics that of benign uterine leiomyoma [1, 2]. Preoperative diagnosis of uterine ESS is critical given the emergence of nonsurgical options for benign leiomyomas, such as uterine artery embolization and high-intensity focused ultrasonography [4]. ESS is classified into high-grade and low-grade subtypes based on morphology, nuclear pleomorphism, and necrosis [5]; the subtypes have different therapeutic and prognostic profiles. High-grade and undifferentiated endometrial sarcoma, referred to as high-grade ESS herein, behaves aggressively and is associated with a 5-year survival rate of only 33% [6, 7], whereas low-grade ESS behaves indolently and is associated with a 5-year survival rate of 91% [7]. Surgery is the primary treatment for both high-grade and low-grade ESS confined to the uterus [8]. For high-grade ESS, adjuvant systemic chemotherapy or radiotherapy is recommended, whereas adjuvant hormone therapy is recommended for low-grade ESS [1]. Pretreatment diagnosis of ESS and differentiation between high-grade and low-grade ESS would assist clinicians in precisely tailoring the treatment plan for patients.

Magnetic resonance (MR) imaging has proven useful in the pretreatment survey of uterine malignancies [9]. With the routine and emerging advanced imaging sequences such as diffusion-weighted (DW), whose measurement were reflected by the biophysical characteristics of the tissue, rendered the discrimination of malignant from benign [10, 11]. More than 60% of benign leiomyomas demonstrate T2 hypointensity as compared with the myometrium [12]. However, varying extent of degeneration and edema or increased cellularity of benign leiomyomas cause T2 hyperintensity, which hinders their distinction from uterine sarcomas [12]. Adding to the diagnostic difficulty is the lack of studies on differentiation between high- and low-grade ESS based on imaging [13–16]. ESS has been reported to manifest as large masses with or without evidence of myometrial invasion on MR images [13], demonstrating T2 hypointense bands [14], marginal nodules and worm-like intra-myometrial nodules [15], or projections in the vessels or along the ligaments [16]. One report showed that high-grade ESS infiltrates the myometrium more destructively than low-grade ESS [5], and another recent report suggested overlapping morphological appearance on MR images [17]. The diagnostic performance of robust MR sequences, namely contrast-enhanced (CE) MR imaging and DW MR imaging with an apparent diffusion coefficient (ADC) value, in differentiation between high- and low-grade ESS remains to be established.

We aim to investigate the diagnostic accuracy of MR imaging in diagnosing and differentiating between high-grade and low-grade ESS.

## Methods

### Study population

**Fig. 1:**
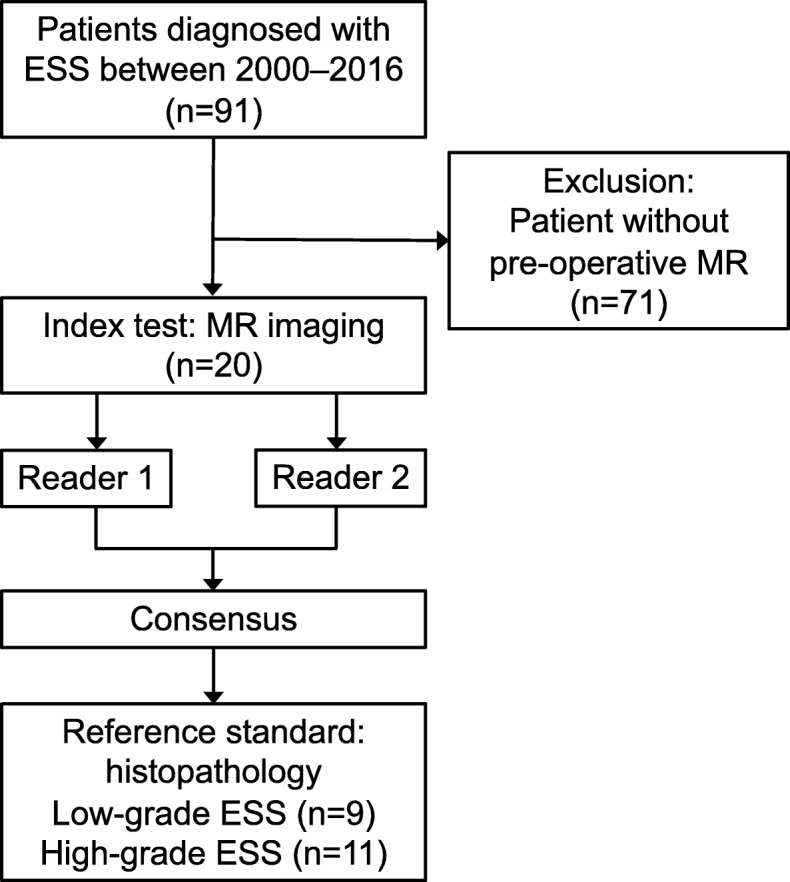
Flow diagram of the study design

Our institutional review board approved this Health Insurance Portability and Accountability Act-compliant retrospective study, and the requirement of informed consent was waived. From a review of our pathology database records between January 2000 and December 2016, we identified 91 cases of pathologically confirmed endometrial stromal and related tumors. Among these cases, MR imaging before hysterectomy or definitive surgery was performed in 34 cases. We excluded 10 cases in which only myomectomy or hysteroscopic removal of the primary tumor for diagnostic purposes was performed without gross residual tumors on MR images. For 4 cases, MR imaging was performed before the introduction of the Picture Archiving and Communication System (PACS); therefore, the images could not be acquired. Finally, 20 cases for which preoperative MR examination was performed and images could be acquired from our current PACS were included in the final analysis. We added the MR images of benign T2-hyperintense leiomyomas excluding hemorrhagic infarctions from a continuous cohort that included 126 benign leiomyomas diagnosed during 2015–2017 in patients who received complete preoperative imaging workup and surgical pathology. Figure 1 shows the flowchart of the study design.

### MR protocol

Given that the study was conducted over 17 years, the MR imaging equipment and scan parameters varied. Of the 20 patients, 10 were examined using a 3 T MR system (6 using Skyra, Trio Tim, Siemens Medical System, Erlangen, Germany; 3 using Trio Tim, Siemens Medical System, Erlangen, Germany; and 1 using Discovery MR750, GE Medical System, Milwaukee, USA) and the rest using a 1.5 T MR system (Magnetom Vision, Siemens Medical System, Erlangen, Germany; Gyroscan Intera, Philips Medical Systems, Best, the Netherlands; Optima MR450w, GE Medical System, Milwaukee, USA; Signa HDxt, GE Medical System, Milwaukee, USA). In general, in the MR scans, phased-array body coils were applied to cover the entire pelvis. The imaging studies were performed during normal respiration and without the administration of premedication or antiperistaltic agents. All studies included T1- and T2-weighted spin-echo sequences and CE MR images (slice thickness, 4 mm; gap, 1 mm; matrix, 256 × 320; field of view, 20 cm). CE MR images was acquired in the sagittal and axial planes at approximately 120–180 s in the equilibrium phase after an intravenous injection with the contrast medium at 0.1 mmol/kg body weight (gadopentetate dimeglumine, Magnevist, Schering, Berlin, Germany), followed by 20-mL saline flush at a rate of 2–3 mL/s. After 2006, DW MR imaging was routinely performed using a single-shot echo-planar technique with fat suppression. ADC maps were generated from isotropic DW images with b-values of 0 and 1000 s/mm^2^ by calculating the slope of the logarithmic decay curve for signal intensity against b-values.

### Imaging analysis

**Fig. 2:**
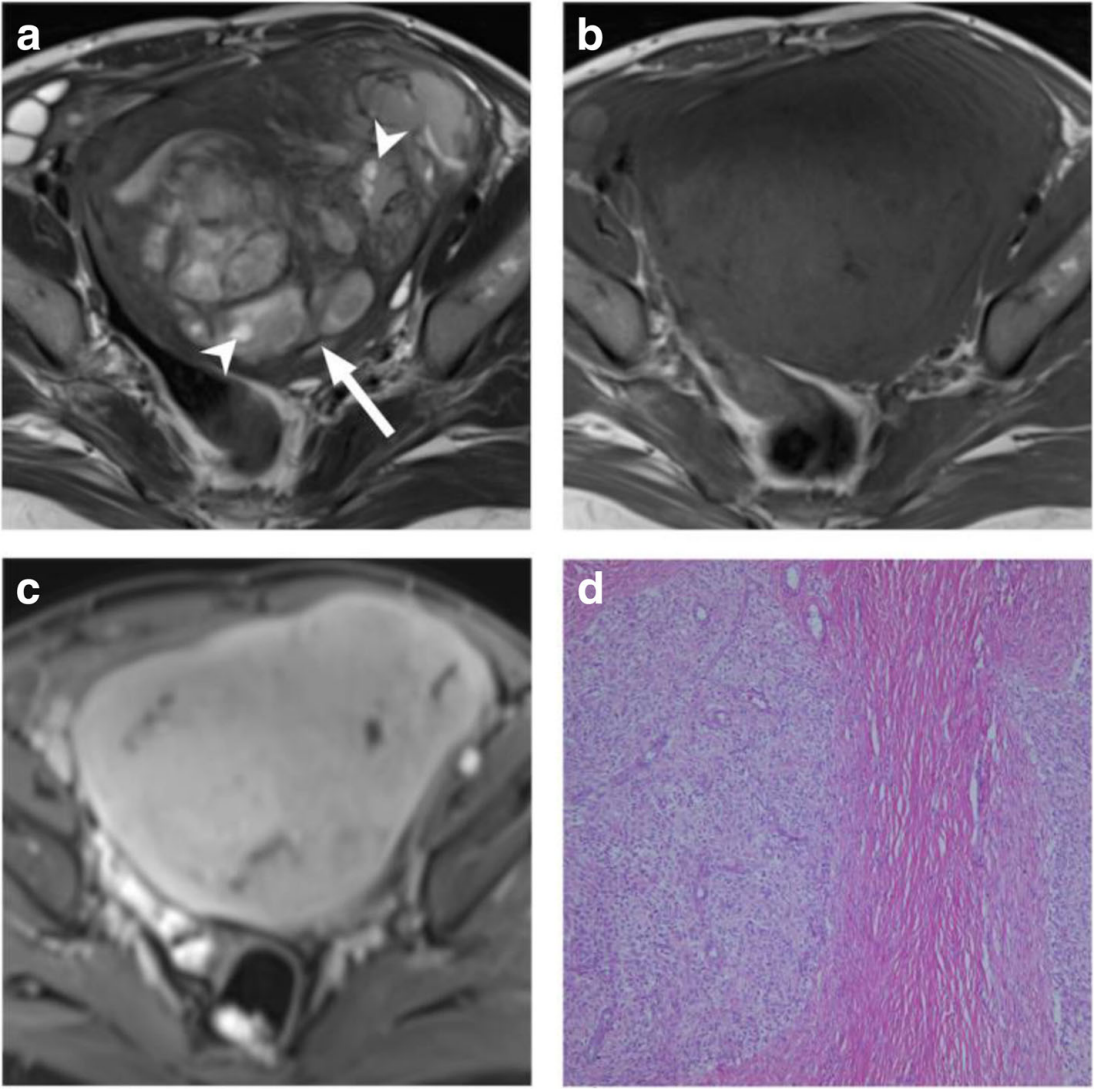
A 35-year-old female patient had a 12.3-cm infiltrative tumor involving both endometrial cavity and myometrium. **a** Axial T2WI image shows tumor signal higher than the adjacent myometrium (arrowhead), with T2 hypointense bands interspersed within the tumor (arrow). **b** This tumor exhibits isointense T1 signal. **c** Post-contrast fat-saturated T1WI shows slightly heterogeneous enhancement. **d** H&E stain shows irregular tumor nests of blue cells permeating the myometrium without an associated stromal reaction. Pathology report yielded a low-grade endometrial stromal sarcoma

**Fig. 3:**
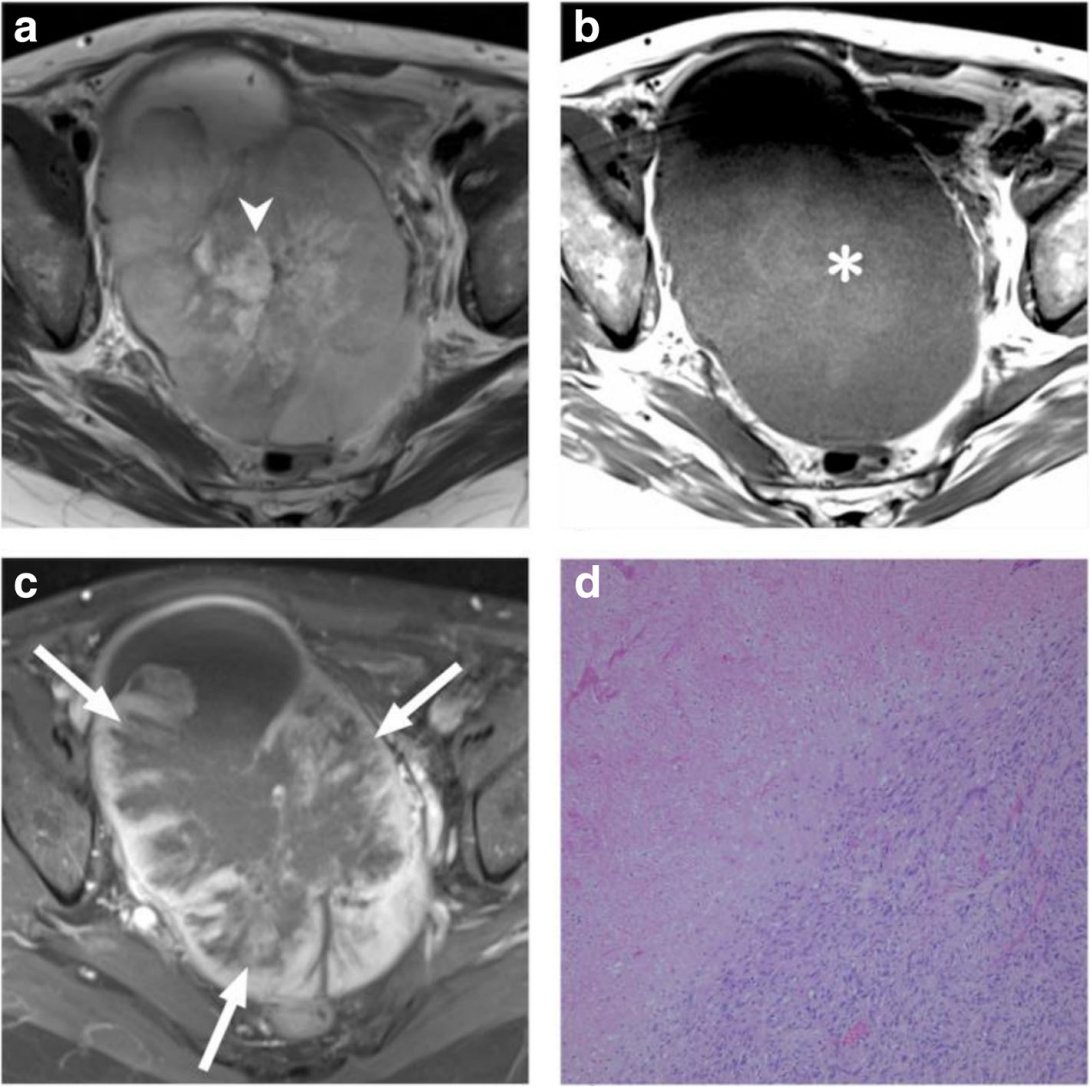
A 61-year-old woman had a 14.5-cm infiltrative tumor involving both endometrial cavity and myometrium. **a** Axial T2WI image shows hyperintensity within the solid tumor representing necrosis (arrowhead). **b** Axial T1WI image demonstrates hyperintensity representing hemorrhage (asterisk). **c** Post-contrast fat-saturated T1WI shows feather-like enhancement (arrow) within the tumor. **d** H&E stain shows small round cells with mitotic figures, as well as extensive necrosis and intratumoral hemorrhage. Pathology report yielded a high-grade endometrial stromal sarcoma

Two radiologists with 12 and 3 years of experience in gynecologic radiology retrospectively and independently interpreted the MR images. All images were stored in the PACS of our hospital and were acquired for analytical purposes. Image review was performed independently using dedicated image processing software (OsiriX MD v8.0.2, Geneva, Switzerland) on an offline macOS system (MacBook Pro, Apple, Cupertino, CA), with the reviewer blinded to clinical information. Two readers independently assessed imaging parameters including tumor size, location, margin, T1 weighting, T2 weighting, CE, and DW, and consensuses on imaging features was achieved through joint review. The worm-like nodular extension was defined as detached or discrete nodules from the primary tumor representing myometrial and lymphovascular invasion [15]. Marginal nodules were defined as nodular lesions at the tumor margin, also representing myometrial invasions of tumors [15]. Intratumoral T2 low-signal-intensity bands were scattered, preserved bundle of the myometrium [14] (Fig. 2). High signal intensities on pre-enhanced T1-weighted images were considered indicators of hemorrhage. We defined necrosis as areas of high signal intensity on T2-weighted images and the lack of enhancement after administration of the contrast medium. The feather-like enhancement was used to describe fine, wispy enhancement interspersed within tumors (Fig. 3). The ADC values of each primary tumor were measured using manually drawn regions of interest on the console with main tumors on the largest tumor plane, excluding the necrotic and nonenhanced portions. The average of the ADC values measured by the two readers independently was considered the representative ADC value for each tumor. Additional file 1: Table S1 details the MR imaging features.

### Histopathologic analysis

The reference standard used in this study was surgical histopathology, which was reviewed by a pathologist with 13 years of experience in gynecologic pathology. The pathological findings including tumor size, differentiation, nuclear atypia, mitotic count, round cell morphology, and necrosis were reviewed and evaluated on hematoxylin–eosin–stained slides. Histopathologic diagnosis was performed based on the 2014 World Health Organization (WHO) classification criteria [18]. The clinical information and MR results were made available to the assessors of the reference standard.

### Statistical analysis

We analyzed the data using the Statistical Package for Social Sciences version 11 (SPSS, Chicago, IL). The Mann–Whitney *U* test and Fisher’s exact test were used to compare the pathological findings and MR imaging features between all ESS and T2-hyperintense leiomyomas and between high-grade and low-grade ESS. A *P* value less than .05 was considered statistically significant. Reader agreement on imaging features and interobserver variability were analyzed using weighted kappa statistics (0.00 ≤ κ <  0.40 indicated poor agreement; 0.40 ≤ κ ≤ 0.70 indicated moderate agreement; 0.70 ≤ κ ≤ 0.90 indicated good agreement; and κ > 0.90 indicated excellent agreement). The overall sensitivity, specificity, and diagnostic accuracy of each imaging characteristic were determined based on the histopathological reference, and the data are represented in 95% confidence intervals. The McNemar test was used to compare the sensitivity, specificity, and diagnostic accuracy of imaging features that showed a significant difference between high-grade and low-grade ESS. Areas under the receiver operating characteristic curve (AUCs) were calculated to compare diagnostic performance in each group.

## Results

### Patients

Eleven patients with high-grade ESS and 9 with low-grade ESS were identified. Table 1 summarizes patient demographics. Patients with high-grade ESS were significantly older than patients with low-grade ESS (median age, 64 vs. 42 years, *P* < .001); however, the ages overlapped substantially. The interval between the MR examination and surgery ranged from 0 to 30 days (median, 11 days). No remarkable adverse events were recorded during MR examination.

**Table 1:** Demographics of the study participants

	T2-hyperintense leiomyoma	High-grade ESS	Low-grade ESS
n	16	11	9
Age (year)			
Median (range)	43 (30–49)	64 (45–78)	42 (29–55)
Menopausal status			
Premenopausal	16 (100.0)	1 (9.1)	8 (88.9)
Postmenopausal	0 (0.0)	10 (90.9)	1 (11.1)
FIGO staging			
I	N/A	5 (45.5)	8 (88.9)
II	N/A	0 (0.0)	0 (0.0)
III	N/A	4 (36.4)	1 (11.1)
IV	N/A	2 (18.2)	0 (0.0)
Treatment			
Surgery alone	16 (100.0)	3 (27.3)	5 (55.6)
Surgery with adjuvant treatment	N/A	7 (63.6)	4 (44.4)
CCRT alone	N/A	1 (9.1)	0 (0.0)

Data in parentheses are percentages

*CCRT* Combined chemoradiotherapy

### MR imaging characteristics of T2-hyperintense leiomyomas and ESS

**Fig. 4:**
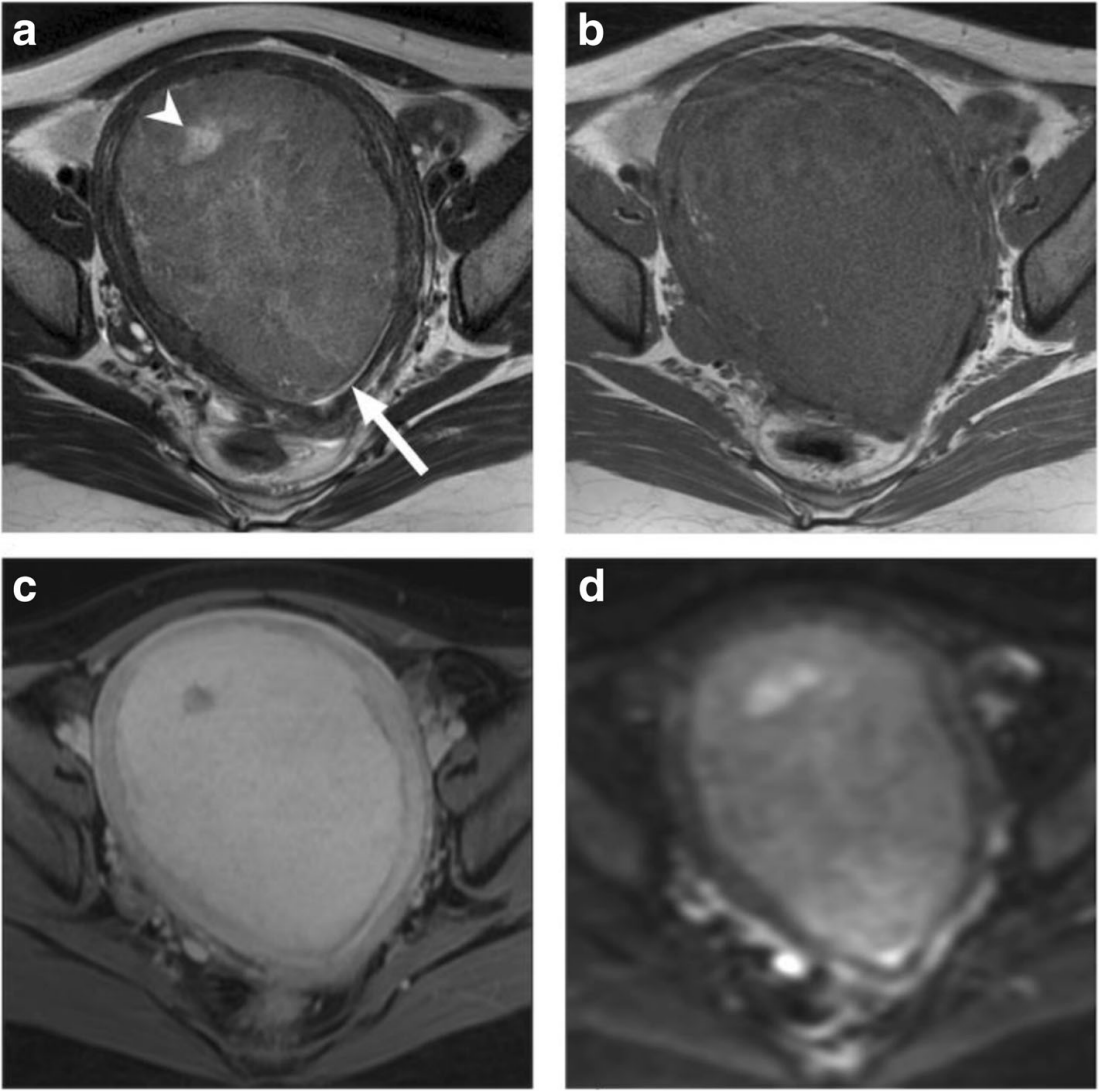
A 48-year-old female had a 12.6-cm intrauterine leiomyoma, arising from the anterior wall of the uterus, compressing the endometrial cavity (arrow). The tumor exhibits higher signal intensity than that of the myometrium on axial T2WI image (**a**), with focus of edema or necrosis (arrowhead), isointensities on axial T1WI image (**b**), equal enhancement as adjacent myometrium on post-contrast fat saturated T1WI (**c**), and mild heterogenous high signal intensity on diffusion-weighted images suggesting diffusion restriction (**d**)

The tumor morphology of pathologically confirmed benign T2-hyperintense leiomyomas differed significantly from that of ESS on MR imaging, as summarized in Table 2. None of the benign leiomyomas demonstrated infiltrative tumor margins, worm-like intramyometrial nodules, marginal nodules, or feather-like enhancement. In contrast to ESS, only a small percentage of leiomyomas showed intralesional necrosis and hemorrhage (*P* = .001 and .002, respectively). Benign leiomyomas showed enhancement that was the same as that in the normal myometrium (Fig. 4), whereas ESS showed less enhancement than that in the adjacent unaffected myometrium (*P* = .001). The ADC value was significantly lower in ESS than in T2-hyperintense leiomyomas (*P* < .001; mean ± standard deviation, 0.99 ± 0.13 × 10^− 3^ mm^2^/s) and low-grade ESS (1.09 ± 0.120 × 10^− 3^ mm^2^/s, *P* < .001).

**Table 2:** MR imaging characteristics between endometrial stromal sarcomas (ESS) and T2-hyperintense leiomyomas

Parameters	ESS	T2-hyperintense leiomyoma	*P* value
n	20	16	
Tumor size (cm)	9.68 ± 3.59	11.66 ± 4.45	0.265
ADC value (× 10^− 3^ mm^2^/sec)	1025.61 ± 130.31	1348.19 ± 162.15	< 0.001 *
Margin			0.014 *
Well-defined	12 (60.0)	16 (100.0)	
Ill-defined	8 (40.0)	0 (0.0)	
Worm-like nodules	8 (40.0)	0 (0.0)	0.014 *
Marginal nodules	14 (70.0)	0 (0.0)	< 0.001 *
Intratumoral nodules	16 (80.0)	7 (43.8)	0.057
T2 hypointense bands	20 (100.0)	1 (6.3)	< 0.001 *
Feather-like enhancement	10 (50.0)	0 (0.0)	0.003 *
Necrosis	13 (65.0)	1 (6.3)	0.001 *
Hemorrhage	14 (70.0)	2 (12.5)	0.002 *
Tumor enhancement			0.001 *
Less than myometrium	15 (75.0)	5 (31.3)	
Equal to myometrium	1 (5.0)	10 (62.5)	
Greater than myometrium	4 (20.0)	1 (6.3)	

Data in parentheses are percentages

*Statistically significant

### MR imaging characteristics of high-grade and low-grade ESS

Table 3 summarizes the MR imaging characteristics for our 20 cases. Reader agreement was good to excellent for tumor size, location, involvement, and MR imaging features. Both high-grade and low-grade ESS appeared as largely solid and occasionally cystic tumors located at the uterine fundus or the body, and they were either confined within the myometrium or involved both the endometrium and myometrium and had demarcated or infiltrative margins. The sizes of high-grade ESS tumors were larger than those of low-grade ESS tumors (11.25 cm^3^ vs. 7.76 cm^3^); however, the difference was not statistically significant (*P* = .056). High-grade and low-grade ESS did not show significant differences in the presence of worm-like, marginal, or intramural nodules. T2 hypointense bands appeared in all high-grade and low-grade ESS tumors. The three imaging features that showed significant differences between high-grade and low-grade ESS tumors were feather-like enhancement (*P* < .001), necrosis (*P* = .017), and hemorrhage (*P* = .050). No significant differences were found in ADC values between high-grade (mean ± standard deviation, 0.99 ± 0.13 × 10^− 3^ mm^2^/s) and low-grade ESS (1.09 ± 0.120 × 10^− 3^ mm^2^/s, *P* = 0.190).

**Table 3:** MR imaging characteristics between high-grade and low-grade endometrial stromal sarcomas

Parameters	High-grade	Low-grade	*P* value	Kappa
n	11	9		
Tumor size (cm)	11.25 ± 2.90	7.76 ± 3.55	0.056	0.828
ADC value (×10^−3^ mm^2^/sec)	0.99 ± 0.13	1.09 ± 0.120	0.190	N/A
Tumor location			0.206	0.971
Fundus	0 (0.0)	3 (33.3)		
Body	5 (45.5)	3 (33.3)		
Cervix	1 (9.1)	0 (0.0)		
Diffuse	5 (45.5)	3 (33.3)		
Tumor involvement			0.336	1.000
Endometrium and myometrium	8 (72.7)	5 (55.6)		
Endometrium only	0 (0.0)	0 (0.0)		
Myometrium only	2 (18.2)	4 (44.4)		
Cervix	1 (9.1)	0 (0.0)		
Serosa penetration	5 (45.5)	1 (11.1)	0.157	0.870
Margin			0.714	0.875
Well-defined	7 (63.6)	5 (55.6)		
Ill-defined	4 (36.4)	4 (44.4)		
Worm-like nodules	4 (36.4)	4 (44.4)	1.000	0.898
Marginal nodules	9 (81.8)	5 (55.6)	0.336	0.875
Intratumoral nodules	10 (90.9)	6 (66.7)	0.285	0.828
T2 hypointense bands	11 (100.0)	9 (100.0)	N/A	1.000
Feather-like enhancement	10 (90.9)	0 (0.0)	< 0.001 *	1.000
Necrosis	10 (90.9)	3 (33.3)	0.017 *	0.780
Hemorrhage	10 (90.9)	4 (44.4)	0.050 *	0.886
Tumor enhancement			0.082	0.920
Less than myometrium	10 (90.9)	4 (44.5)		
Equal to myometrium	1 (9.1)	3 (33.3)		
Greater than myometrium	0 (0.0)	2 (22.2)		

Data in parentheses are percentages

*Statistically significant

### Diagnostic accuracy of the characteristics in differentiating between high- and low-grade ESS

**Fig. 5:**
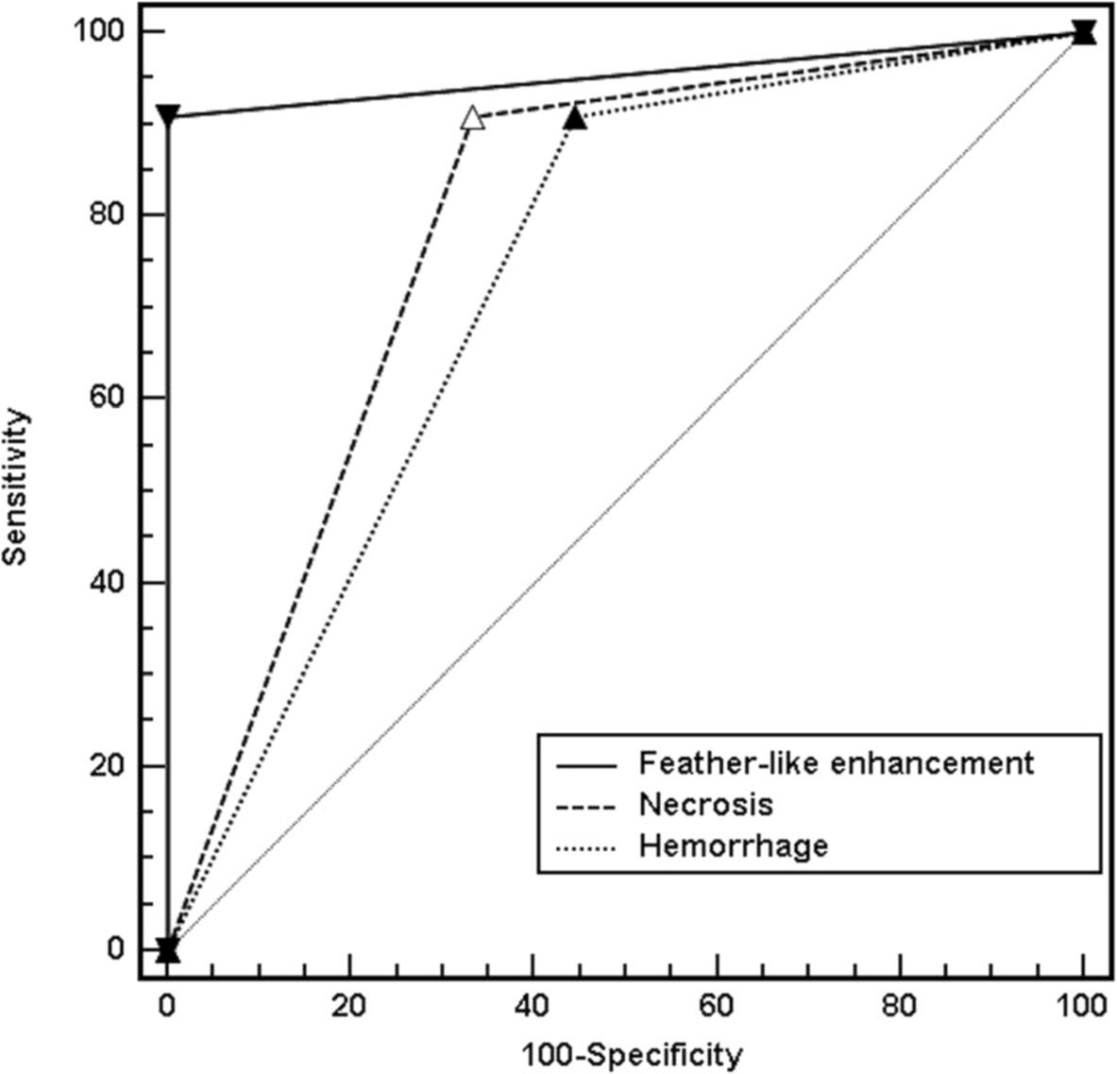
Areas under the receiver operating characteristic curve (AUC) analysis to compare diagnostic performances. Feather-like enhancement demonstrated an AUC of 0.955, significantly higher than that of necrosis (0.788) or hemorrhage (0.732, *P* = 0.045), in differentiating low-grade from high-grade endometrial stromal sarcoma (ESS)

We tested the diagnostic accuracy of the three outstanding imaging features: feather-like enhancement pattern, necrosis, and hemorrhage (Table 4). The feather-like enhancement yielded a diagnostic accuracy of 95% in differentiating between high-grade and low-grade ESS; this feature yielded significantly superior differentiation than necrosis (80%, *P* = .033) or hemorrhage (75%, *P* = .007). It had an AUC of 0.955, which was significantly superior to that of necrosis (0.788) or hemorrhage (0.732, *P* = .045) (Fig. 5).

**Table 4:** Diagnostic accuracy in differentiating between high-grade and low-grade endometrial stromal sarcomas based on selected MR imaging features

Parameters	n	TP	TN	FP	FN	Sensitivity	Specificity	Accuracy	PPV	NPV
Feather-like enhancement	20	10	9	0	1	90.9 (58.7–99.8)	100 (100–100)	95.0 (75.1–99.9)	100 (100–100)	90.0 (55.5–99.7)
Reader 1	20	10	9	0	1	90.9 (58.7–99.8)	100 (100–100)	95.0 (75.1–99.9)	100 (100–100)	90.0 (55.5–99.7)
Reader 2	20	10	9	0	1	90.9 (58.7–99.8)	100 (100–100)	95.0 (75.1–99.9)	100 (100–100)	90.0 (55.5–99.7)
Necrosis	20	10	6	3	1	90.9 (58.7–99.8)	66.7 (29.9–92.5)	80.0 (56.3–94.3)	76.9 (46.2–95.0)	85.7 (42.1–99.6)
Reader 1	20	10	6	3	1	90.9 (58.7–99.8)	66.7 (29.9–92.5) *	80.0 (56.3–94.3) *	76.9 (46.2–95.0)	85.7 (42.1–99.6)
Reader 2	20	10	6	3	1	90.9 (58.7–99.8)	66.7 (29.9–92.5) *	80.0 (56.3–94.3) *	76.9 (46.2–95.0)	85.7 (42.1–99.6)
Hemorrhage	20	10	5	4	1	90.9 (58.7–99.8)	55.6 (21.2–86.3)	75.0 (50.9–91.3)	71.4 (41.9–91.6)	83.3 (35.9–99.6)
Reader 1	20	10	5	4	1	90.9 (58.7–99.8)	55.6 (21.2–86.3) *	75.0 (50.9–91.3) *	71.4 (41.9–91.6)	83.3 (35.9–99.6)
Reader 2	20	9	5	4	2	81.8 (48.2–97.7)	55.6 (21.2–86.3) *	70.0 (45.7–88.1) *	69.2 (38.6–90.9)	71.4 (29.0–96.3)

Data in parentheses are 95% confidence intervals

*TP* True positive, *TN* True negative, *FP* False positive, *FN* False negative, *PPV* Positive predictive value, *NPV* Negative predictive value

*, *P* < .05 McNemar test, as compared with the feather-like enhancement

## Discussion

In the present study, we found that the presence of the feather-like enhancement was the most accurate MR imaging characteristic of high-grade ESS, and this feature was absent in low-grade ESS. CE MR imaging yielded significantly higher diagnostic accuracy and specificity in differentiating between high-grade and low-grade ESS compared with hemorrhage on T1-weighted or necrosis on T2-weighted MR imaging. The excellent agreement between junior and senior readers supported CE MR imaging as an objective method for preoperative MR evaluation. The feather-like enhancement likely indicated destructive myometrial invasion in high-grade ESS in contrast to the finger-like invasive projections in low-grade ESS [19]. In addition, CE MR imaging can be useful in distinguishing ESS from other uterine sarcomas. Uterine leiomyosarcomas or smooth muscle tumors with uncertain malignant potential (STUMP) exhibit pocket-like central nonenhanced areas that correlate with areas of coagulative necrosis [4, 20]. By contrast, carcinosarcomas demonstrate prolonged tumoral enhancement exceeding that of the myometrium [21–24]. Leiomyomas with hyaline degeneration contain numerous bands of hyalinized collagen interlacing viable cells and the necrotic focus to support the structure [25], and entire leiomyoma tumors exhibit scattered nonenhanced areas on CE MR imaging [4]. The feather-like enhancement was found in all cases in published data [13, 15, 26–28], as summarized in Table 5. Despite the rarity of ESS, we managed to recruit the largest patient cohort to date. To the best of our knowledge, no previous study has been focusing on the pre-treatment differentiation of low-grade and high-grade ESS using MR imaging.

**Table 5:** Literature summary of MR imaging findings of high-grade and low-grade endometrial stromal sarcomas

Author	Year	Patient number (Age, years)		Size (cm)	Feather-like enhancement		Necrosis		Hemorrhage		Reference
		HG	LG		HG	LG	HG	LG	HG	LG	
Koyama	1999	2 (50, 64)	6 (20–49)	3–18	N/A	–	+/−	–	+/−	–	[14]
La Fianza	1999	1 (55)	0	11	+	N/A	+	N/A	+	N/A	[26]
Ueda	2000	1 (53)	1 (47)	8.5, 9	+	–	+	–	N/A	–	[27]
Gandolfo	2000	1 (52)	1 (69)	10, 10	+	N/A	+	+	+	+	[13]
Ueda	2001	4 (35–55)	4 (53–66)	1–19	+	–	+/−	+/−	+/−	+/−	[15]
Toprak	2004	0	1 (24)	N/A	N/A	–	N/A	–	N/A	–	[40]
Chien	2005	0	1 (46)	N/A	N/A	–	N/A	–	N/A	–	[41]
Kusaka	2006	1 (39)	0	8	N/A	N/A	+	N/A	+	N/A	[42]
Hayasaka	2006	1 (74)	0	N/A	+	N/A	+	N/A	+	N/A	[28]
Tamai	2008	0	2 (40, 47)	5, 10	N/A	N/A	N/A	–	N/A	–	[16]
Fujii	2010	0	2 (44, 53)	N/A	N/A	–	N/A	–	N/A	–	[39]
Furukawa	2010	0	3 (20–57)	5.5–11	N/A	–	N/A	–	N/A	+/−	[29]
Liu	2015	1 (73)	0	5.2	N/A	N/A	+	N/A	+	N/A	[43]

+ = present, − = absent, N*/A* Not available, *HG* High-grade, *LG* Low-grade

The T2 hypointense band is a common MR imaging feature for both high-grade and low-grade ESS; it represents preserved myometrial bundles separated by clusters of tumor cells permeating the myometrium [14]. Furukawa et al. described a T2 low-signal-intensity rim that might be the fibrous tissue layer located between viable tumor cells and the normal myometrium [29], which was absent in our imaging studies. Notably, intratumoral T2 hyperintensity is characteristic not only of ESS but also of benign degenerative leiomyomas, leiomyosarcomas or STUMP [30], and carcinosarcomas [21–23]. The T1-weighted hyperintensity observed in high-grade ESS, which represents hemorrhage [19], has also been observed in leiomyosarcomas or STUMP [20] and carcinosarcomas [23, 24]. The worm-like or marginal nodules in ESS were once considered invasive features representing intra-lymphatic or intravascular involvement [15]; however, in our study, they appeared in both high-grade and low-grade ESS and showed no statistically significant difference between the two groups. ESS could show great vessel invasion into the inferior vena cava, heart or pulmonary vessels [31–34], but only one of our cases demonstrated uterine vein invasion on MR imaging. This may be because the cohort included early stage cases.

DW imaging helps in the differentiation of malignant from benign in both pre- and post-treatment imaging studies [35]. The post-treatment follow-up using advanced diffusion-weighted imaging modules in gynecological oncologic cancers is a novel idea that has not been well studied yet. This article focuses mainly on the pre-treatment diagnosis and how the pre-treatment imaging can help to guide the clinical decision making in treatment.

Our study showed that tumor ADC values were significantly lower in ESS than in T2-hyperintense leiomyomas but were not significantly different between high-grade and low-grade ESS. Histopathologically, high-grade ESS is characterized by marked cytological atypia, nuclear pleomorphism, high mitotic activity, and extensive invasion of sarcomatous components [36], whereas low-grade ESS is characterized by densely uniform stromal cells with minimal cellular pleomorphism and mild nuclear atypia [5]. The ADC values in ESS are influenced by the nuclear-to-cytoplasm ratio and cellular density in both stromal and sarcoma components [16, 37]. Hence, the ADC values of ESS are not indicative of the aggressiveness of ESS. The results of our study are in line with those of other studies on uterine sarcomas such as leiomyosarcomas or STUMP [4], carcinosarcomas [24, 38], and ESS [16, 39], which also showed lower ADC values for ESS than for the myometrium.

This study had limitations. First, this was a retrospective study. In addition, given the long 17-year study span, the MR scanners and parameters varied. Second, during the study period, three ESS classification guidelines were applied, and we adapted the most recent 2014 WHO classification [18]. The actual frequency and clinical features of the new category, that is high-grade ESS, are unknown. Future studies should apply the new WHO classification system to determine clinical, imaging, pathological, and molecular differences between low-grade ESS, high-grade ESS, and undifferentiated uterine sarcomas to understand the prognostic significance of MR imaging.

## Conclusions

DW MR imaging is useful in diagnosing ESS against T2-hyperintense leiomyomas, whereas contrast enhancement aids in further differentiating between high- and low-grade ESS. Pretreatment differentiation between high-grade and low-grade ESS based on MR imaging would assist clinicians in selecting the most appropriate treatment plan.

## Additional file

Additional file 1: **Table S1.** List of MR imaging features analyzed in the present study. (DOCX 18 kb)

## Data Availability

Not applicable.

## Abbreviations

ADC: Apparent diffusion coefficient
AUC: Areas under the receiver operating characteristic curve
CE: Contrast-enhanced
DW: Diffusion-weighted
ESS: Endometrial stromal sarcoma
MR: Magnetic resonance
PACS: Picture archiving and communication system
STUMP: Smooth muscle tumors with uncertain malignant potential
WHO: World Health Organization
