# Supplementary material for: Utility of diffusion-weighted and contrast-enhanced magnetic resonance imaging in diagnosing and differentiating between high- and low-grade uterine endometrial stromal sarcoma
Source: Cancer Imaging. 2019 Sep 12;19:63. doi: 10.1186/s40644-019-0247-z (PMC6739916; doi:10.1186/s40644-019-0247-z)
Supplement: Supplementary file 1 — Table S1. List of MR imaging features analyzed in the present study. (DOCX 18 kb) [file 40644_2019_247_MOESM1_ESM.docx]

**Additional file 1; Table S1.** List of MR imaging features analyzed in the present study

| Features | Description | Reference |
| --- | --- | --- |
| Worm-like nodules | Intramyometrial serpentine nodular extension, detached from the tumor. | [[13](#_ENREF_13)] |
| Marginal nodules | Nodular lesion at tumor margin, attached to the tumor margin. | [[13](#_ENREF_13)] |
| Intratumoral nodules | Multiple nodule formation inside the tumor. | [[13](#_ENREF_13)] |
| T2 hypointense bands | Bands of low SI on T2WI throughout areas of myometrial involvement of tumor. | [[12](#_ENREF_12)] |
| Feather-like enhancement | Fine, feathery enhancement in the early/late contrast enhancement. | - |
| Necrosis | High SI on T2WI and lack of enhancement after contrast medium administration. | - |
| Hemorrhage | High SI on T1WI. | [[13](#_ENREF_13)] |

Note—SI = signal intensity. T1WI = T1-weighted images. T2WI = T2-weighted images.
